# Book Reviews

**Published:** 1827-03

**Authors:** 


*August* 1826.

### CRITICAL ANALYSES.

Quæ laudanda forent, et quæ culpanda, vicissim

Illa, prius, cretâ; mox hæc, carbone, *notamus.*—Persius.

*Appendix to the Papers on the Nerves, republished from the Royal Society's Transactions, by* Charles Bell: *containing Consultations and Cases illustrative of the Facts announced in these Papers.*—8vo. Longman and Co. London.

Since the announcement in the Philosophical Transactions of these discoveries, we have promulgated them by such means as were in our power, because we foresaw that they would claim the attention of the profession in all countries; and we have now the satisfaction of thinking that we were among the first, if not the very first, to proclaim their importance.

If we look back to the labours of preceding physiologists on the Nervous System, we shall find that little was accomplished by them, in ascertaining with precision the distinct functions of individual nerves. Hence the classification of nervous disorders by systematic authors have been unsatisfactory, being formed in ignorance of the uses of the nerves. For the same reason, physicians have hesitated to confide in their knowledge of the nerves, while attempting to discriminate between diseases; and this department of anatomy has generally been shunned as being too obscure to afford a hope that practical benefits could be derived from its study. But a more favourable prospect has been held out to us for some time, by the views of our author. The Cases and Observations which are before us, in addition to those already published, afford many proofs of the advantages which have resulted from his investigations.

The first set of Cases are illustrative of the first paper read before the Royal Society, wherein it was shown that the fifth pair of nerves bestows sensibility to the head, and power of motion to the muscles of the jaws; and that the portio dura controls the actions of certain muscles of the face, and is connected in a particular manner with the functions of respiration. It was also shown that these two nerves belong to distinct classes.

It may, perhaps, be recollected by our readers that Mr. Bell selected these as the subjects of his first paper, in order to exhibit the distinctions between nerves supplying the same parts. He stated, that he despaired of exciting attention to the distinction in the nerves of the throat and chest, from those parts being more complicated in their anatomy and the number of their functions. We have a proof in this Appendix of the correctness of the author's view of this matter; for we find all the cases which have been supplied by others are of affections of the nerves of the face: the cases of disorders in the nerves of the neck and chest are from his own observation. He expresses his conviction, however, that, when practitioners shall have a distinct knowledge of the anatomy of the respiratory nerves, cases illustrative of their functions will accumulate.

We have attended from time to time Mr. Bell's Lectures upon the Nervous System, both at Windmill-street and at the College of Surgeons, and we must confess that the subject has appeared to possess still more interest, and to be more easily comprehended, in his Lectures than in his Essays. The course of observations by which he was led to distinguish the nerves into classes, appears to have been this:—Viewing the regularity with which certain nerves arise from continued tracts of nervous matter, and contrasting with these the irregularity of others, he endeavoured to ascertain, by experiments on the roots of the spinal nerves, whether those nerves which arise from the same tracts possess similar endowments. He proved that the anterior roots bestow muscular motion; and, tracing upwards the column of medullary matter from which these anterior roots arise, he observed the ninth pair, the sixth, and the third, coming as it were from the same nervous tract. The distribution of these proved to him that they also are motor nerves. Having in his experiments divided the roots of the spinal nerves, and satisfied himself that the anterior bestow the power of motion, he was at the same time convinced that sensibility resulted from the posterior roots; because he observed that there was no convulsion of the muscles when they were irritated. But the violence necessarily used in making these experiments prevented him from judging of the degree of sensibility which remained after having cut the posterior roots. Besides, it was then the commonly received opinion that nerves with ganglia upon them are insensible. Considering himself compelled to prove more satisfactorily to others this important fact—that nerves with ganglia may possess sensibility, he turned his attention to the head, in order to find which was the nerve of sensation there. He found the fifth pair, which has a ganglion on one of its roots, and is almost universally distributed over the head. There is also another nerve—viz. the portio dura, supplying a considerable portion of the head, but it has no ganglion at its origin. The question to be decided, therefore, was, which of these two nerves bestowed sensibility?—whether the fifth pair, which has a ganglion, or the portio dura, which has not? Before venturing to make any experiment, he contemplated the subject, through the medium of anatomy, still more closely, and he perceived that the fifth pair corresponded with the nerves of the spine: it resembled them in having two roots, and a ganglion upon one of them. If, then, his conjecture was correct, this nerve must be of a compound nature: the source of sensibility to the head, and also a nerve bestowing voluntary motion.

The question now arose, what might be the use of the portio dura, which went to some of the parts which were already supplied by the fifth pair? In what respects are the muscles of the face peculiar, that they should possess this additional nerve, differing from the fifth pair in its anatomical character? He studied the muscles of the face with great care, and discovered that they have many peculiarities: in particular, that they are related very closely with the operations of the organ of respiration. He remarked that the portio dura arises at a distance, and takes a circuitous course to the muscles, (in which it differs from the spinal nerves, or fifth pair, whose branches pass directly to their destination,) and that its origin is close to that of the great nerve, which supplies the lungs.

He observed a similar peculiarity in the course and distribution of another nerve,—viz. the spinal accessory; the roots of which arise from the cervical portion of the spinal marrow, and the distribution of which is to the sterno-cleido-mastoideus and trapezius: yet it does not, like the common cervical nerves, take a transverse short course to these muscles; but it rises in the first place into the skull, that it may emerge from it again in connexion with the nerve of the lungs. Examining now the actions of those muscles on which the spinal accessory is distributed, he discovered that, besides possessing the usual actions of voluntary muscles, they are combined in a peculiar manner with the apparatus of breathing. Thus, by viewing the anatomy without having recourse to experiments, which, if made prematurely, might have disturbed his course of reasoning, he was led to consider that the fifth pair is the spinal nerve of the head, and that the portio dura commands certain actions of the muscles of the face, which are particularly connected with respiration.

The experiments on the fifth nerve and the portio dura have proved that his views of the functions of these nerves were correct. When the fifth pair was divided, sensibility was destroyed; thus proving that a ganglionic nerve possesses sensibility, and also removing any doubt of the posterior roots of the spinal nerves being capable of bestowing sensation. It was likewise proved, by irritating and dividing this nerve, that it regulates the motions of the jaws.

Thus our author succeeded in proving that, in its origin, distribution, and function, the fifth pair resembles the nerves of the spine; and it may be perceived how much assistance this afforded him in ascertaining precisely the functions of the other nerves. He experimented on the portio dura, and then upon the spinal accessory; and the results confirmed him in his opinion that they were connected with the organ of respiration, and superadded for this peculiar purpose. Afterwards he was enabled to add other nerves to this particular class.

It is not our intention to enter upon the arguments by which he has supported these views; but we observe that the idea of his classification originated from having first discovered the functions of the roots of the spinal nerves, and we find that he applied to the portio dura the name *respiratory nerve of the face*, after having ascertained that the fifth pair resembles the spinal nerves. It was from having discovered that there is a "symmetrical" system of nerves, including those of the spine and the fifth pair, common to all creatures, whatever may be the apparatus by which their respiration is performed, that he was led to investigate the organ of respiration in man and other animals, to ascertain the functions of certain other nerves, which are "irregular."

Our author has repeatedly asserted that he owes these views to anatomical, rather than to experimental investigations. In this Appendix he supplies us with several instances of the want of success attending the researches of those who are mere experimenters, even when they have had an opportunity of seeing the phenomena on the human body. After relating a case in which it was proposed to Mr. Bell that he should divide the portio dura, he states what would have been the consequences of such an operation: "His eye would have remained open; for the attollens palpebræ being supplied by the third nerve, and the orbicularis palpebrarum by the seventh, the cutting of the latter would have paralysed the eyelids; they would have remained open, the eye would have become inflamed, and probably opaque; there would have been greater deformity, and blindness to boot." An eminent surgeon in London, (p. 67,) as well as Mr. JOHN BELL and others, have extirpated tumors from the angle of the jaw, and thus divided the portio dura: paralysis of the muscles of the face was the consequence in all these instances. Again, surgeons have frequently divided the branches of the fifth pair for tic douloureux, and no paralysis of the muscles has resulted from these operations. Mr. Bell is thus led to remark, "that if the portio dura has been repeatedly cut, and also the branches of the fifth pair, without a conception arising in the operator's mind of the functions of these nerves, it brings us forcibly to the conclusion that it is through the knowledge of the anatomy, and not by what is termed experience, that we are to obtain correct notions of the functions of parts, and more especially of the nerves." It would appear that Mr. Bell has not consulted Dr. DARWIN's Zoonomia, for we find there a striking illustration of his opinion. A gentleman, having tic douloureux, was under the care of three eminent practitioners—Dr. Darwin, Mr. Cruickshanks, and Mr. Thomas. Nine incisions, "together with some smaller ones," were made on the left side of his face: every nerve of that side of the face, including branches of the fifth pair and the seventh, were divided, yet there is not one word concerning the defect of sensation or of motion: "he set out for Leicestershire perfectly restored."

In pursuing his investigations, we may perceive that our author has been led to his discoveries by adhering to the principle stated in his first paper,—viz. that "the nerves of the animal frame are complex in proportion to the variety of functions which the parts have to maintain." Being convinced that there is a coincidence between the number of nerves transmitted to an organ and the compound nature of its functions, he directed his first attempts to distinguish what are the different combinations into which the parts enter; then he reflected upon the origins and distribution of the various nerves, to ascertain which were most likely to bestow the separate endowments. His experiments have been resorted to, not as the direct means of discovery, but to verify conclusions drawn from the contemplation of the structure of the body. We regard the discovery of a new motion of the eye, which is frequently referred to in these "Cases," as affording a remarkable proof of the advantages of investigating the functions of parts by the study of their anatomy.

In many courses of his public lectures, Mr. Bell was accustomed to express his difficulties about the function of the fourth nerve: he used to hold out the attempt to discover its use as a good subject for his pupils on which to exercise their ingenuity. The train of observations by which he overcame the difficulty appears to have been nearly this: The question to be solved was the cause of the superior muscle being supplied by the fourth nerve. He contrasted the recti muscles, which are under the influence of the third, with the obliqui, one of which is supplied by a branch of the fifth and the other by the fourth nerve. He found that the recti are sufficient for performing all the voluntary movements of the eye; and he was of opinion that the obliqui are not adapted to act in association with the straight muscles, on account of the oblique direction of their attachments. He was thus induced to look for some other use for the oblique muscles. The circumstance of the fourth nerve arising at a distance near the portio dura, seemed to point out some connexion between the action of the superior oblique and the closing of the eyelids. The question then arose, is there any change in the position of the eye when the eyelids are closed? This admitted of an easy experiment: desiring a friend to attempt to shut his eyelids whilst he held them open with his fingers, he observed the cornea was tilted upwards. This position of the eye pointed out the action either of the superior rectus or of the inferior oblique muscle. He now made an experiment on the eye of a monkey: he cut through the insertion of the superior rectus, yet the eye rolled upwards whenever the animal was threatened; thus proving that the motion he observed was produced by the action of the inferior oblique muscle. He came finally to this conclusion, that, while the eye is under the control of the recti, the oblique muscles balance each other, the superior oblique being in a state of opposite action to the inferior: but this condition ceases when we wink, or when the eye is removed from the command of the voluntary muscles during insensibility, as in sleeping, fainting, &c.—then the superior oblique relaxes, and the inferior turns up the eye. This led him to comprehend why the fourth nerve (that of the superior oblique muscle,) does not arise from the nearer part of the brain, as the third, but is to be traced back to the point of origin of the seventh. He concluded that the eyelid and the eyeball sympathise in their motions by the relation of these nerves at their roots.

We have considered that it would be interesting to our readers thus to point out how these discoveries have resulted from examining and reasoning upon the anatomy. Our author has conducted his investigations by contemplating the structure of the body, and thence inferring the uses of the individual parts: afterwards he submitted his suggestions to the test of experiments, which on this account have been "few and simple."

Of the Cases in the Appendix there are nineteen, which illustrate affections of the portio dura. The following facts are pointed out by them: 1st. It is shown that practitioners have been alarmed with the apprehension of apoplexy, in cases where the paralysis has depended merely upon an affection of the portio dura. 2dly. That motion of the side of the face may be destroyed without the sensibility being diminished, or the motions of the jaw affected. 3dly. That cutting branches of the portio dura in operations upon the face is attended with distortion of the countenance, and perhaps blindness. 4thly. That suppuration within the ear, by affecting the portio dura, may produce paralysis. 5thly. That suppuration before the ear, and under the angle of the jaw, may be attended with similar consequences. 6thly. That paralysis of the face may occur from an accident by which the temporal bone is injured. 7thly. That, by pressure of tumors on the fifth pair, the sensibility of a part of the face may be lost; by pressure on the seventh pair, the motions of the muscles of the face may be destroyed. 8thly. When patients cannot close the eyelids, the eyeball may be seen revolving upwards when they make the attempt. 9thly. The cornea is observed permanently turned up during sleep. 10thly. The eyelids being paralysed, some patients employ the fingers to close them occasionally. 11thly. One patient, from paralysis of the muscles of his nostril, was obliged to pull it open with his finger in order to breathe freely. 12thly. The muscles of the face participate in spasmodic twitchings, which affected the muscles of inspiration.

A woman was seen during the violent efforts of labour, one side of her face remaining motionless; " and the countenance assumed a singularly ludicrous aspect."

A patient was seen dying: one side of the face continued placid, whilst the mouth and nostrils were convulsively pulled towards the other side, " producing a frightful expression of countenance."

At p. 34, there is a case of paralysis of all the voluntary muscles of the eyeball, except the abducens. The eye was seen to roll up during the act of winking. This is followed by a note referring to an incessant tremulous motion of the eye, in a patient who had lost one of his eyes, and had an opaque spot on the cornea of the other. He was insensible of this movement of the eye, and saw objects naturally and at rest. Mr. Bell asks, " was this a defect arising from disorder of the nerves, or an accommodated action to the opacity of the centre of the cornea?" We have seen a case precisely similar to the above: A girl, named Sophia Walker, æt. seventeen, has an incessant motion in both her eyes. She lost her left eye during infancy: it is now shrunk. On the inside of the cornea of the right eye, there is a leucomatous spot, nearly opposite the pupil: the iris adheres to the cornea at this part. The motion of the eye is from angle to angle, and is performed rapidly and incessantly. Her mother says it has continued so ever since her infancy. The girl is unconscious of her eye moving: when she looks at an object, whether near or at a distance, the object appears at rest; she can thread her needle easily. When she reads, there is no cessation of the motion; when she looks at her face in the glass, she can see her eye moving. When asked to strain to look outwards or upwards, still the motion continues. When the eyelids are held apart, and she is desired to make an effort to close them, the cornea revolves upwards so as to be almost hid: the eyeball remains fixed in this position. On her closing the eye gently, the prominent cornea is seen moving opposite to where the eyelids meet. The mother was directed to observe this motion communicated to the eyelids when the eye was closed, and to look whether it continued during sleep. Three days afterwards, she informed us that there was no motion whatever in the eye when her daughter was asleep.—A child, about four years old, has always been very near-sighted. Both eyes have a continual rapid motion, which is commonly in a transverse direction, but sometimes they converge so as to produce the appearance of squinting. When an object was placed so close that the child could see it, the eyes became stationary. It appeared as if the attention of the child required to be fixed upon an object before the motion ceased. This wandering of the eye resembled what we have seen in children with congenital cataract.

We give these cases to show the difficulty of accounting for the phenomena, and we make no attempt to explain them. But we would recommend Mr. Bell to bestow some attention upon these two cases, as they appear to be intimately connected with his observations on the motions of the eye, and may perhaps conduct him to some conclusion.

At p. 79, there is a case of Spasmodic Affection of the Muscles of the Jaw: the author contrasts this with the affections of the portio dura, and states his opinion that the fifth pair is in this instance the nerve affected. The cases which follow illustrate affections of the nerves of the throat, neck, and chest. The first is an instance of complete paralysis of the voluntary muscles of the tongue: the author remarks, "it is evident that she can swallow, from her surviving the attack, which circumstance declares the glosso-pharyngeal nerve in activity; and we are told that she had the taste and the natural feeling of the tongue: that is, the function of the fifth pair was entire." He therefore concludes that the ninth pair was affected:—when he divided that nerve in a dog, the consequences nearly resembled those described in the above case. The author informs us he attended at the same time a young lady who could not swallow, and a boy who had entirely lost his speech. He relates the latter case at p. 87: this boy appears to have had disease of the temporal bone, which affected the nerves at the base of the brain. The most striking circumstances were, that he could readily make various noises, as in hollowing or laughing: he could masticate and swallow, and he could twist his tongue out of his mouth to either side; but, while he was making the effort to speak, there could not be discovered the slightest motion in his larynx. About two years afterwards he suddenly recovered his speech, by the bursting out of matter, as if from the Eustachian tube. Mr. Bell imagines that the inflammation had disturbed the operations of the nerves, without altogether destroying their influence: deranging, for instance, the fine associations necessary to speech, without arresting the motions of the tongue.

The remaining cases are more particularly illustrative of the distinct affections of the respiratory and symmetrical systems of nerves of the neck and chest. The author introduces them by referring to cases of hemiplegia, as affording opportunities of distinguishing an act of respiration from a simple voluntary action. "Although the patient cannot, by a direct effort of the will, move the muscles of one side of the neck or of the shoulder, yet, when he draws the breath, coughs, sneezes, or yawns, these muscles are put into action. A note from Dr. Abercrombie, addressed to Mr. Shaw, (p. 120,) illustrates this circumstance: A hemiplegic patient was much affected with yawning, and every time he yawned the paralytic arm was raised up with a firm steady motion. Our readers must have remarked, while observing difficult breathing, that there are many muscles of the neck, besides others arising from the shoulders, which are necessary for respiration. The head and shoulders become fixed, to allow the muscles arising from these points, and inserted into the chest, to act with greater force. But, even in easy breathing, the mastoideus acts in raising the thorax, the shoulders are elevated, and the serratus anticus expands the margins of the chest: it will be found that these actions are as necessary as the contraction of the diaphragm. It was from the author having remarked that a peculiar class of nerves is distributed to the muscles which perform these actions, that he has applied to them the name Respiratory.

At p. 102, there is a case of Spasmodic Twitchings, such as are often found in the muscles of the face: they were continued down upon the side of the neck, shoulder, and chest, being confined to the muscles of inspiration. This is succeeded by another case of a more distressing kind—a spasmodic affection of the face, throat, shoulders, and chest, on both sides of the body, which continued without any intermission, and was subject to be aggravated by excitement. Here the same class of muscles,—viz. those of inspiration, were deranged: the voluntary movements of these muscles were retained.

The next case is an affection of the Spinal Accessory Nerve, which at intervals produced violent actions in the sterno-cleido mastoideus and trapezius, so that the patient's "ear approached the sternum, and the chin was pitched upwards." He could turn his head in all directions, except at the time he was thus seized. This is followed by another case of spasmodic affection of the muscles of the neck, which produces a continual rolling motion of the patient's head, so that "it turns twenty-two times in the minute." The case that succeeds this is a spasmodic contortion of the head and neck, produced by the violent action of the sterno-cleido mastoideus and trapezius, which dragged the patient's head completely down to the shoulder, and was attended with considerable pain: no support or control by bandage could be borne.

Our author terminates his cases by noticing some affections of the Spine, which afford examples of the act of respiration being continued through the influence of the class of nerves which he calls respiratory, when the "symmetrical" system is destroyed by paralysis. An interesting case is narrated of a boy, who had disease of the upper part of the cervical vertebræ, producing paraplegia. The abdominal muscles were totally inactive, and remarkably wasted, so that they were seen rising and falling according to the distention and contraction of the intestinal canal. His breathing was performed with great difficulty, and at the same time his voice became feeble. The sterno-cleido mastoideus was observed to be in strong action. When he attempted to cough, he raised his chest, but he could give no impulse in discharging the air: he performed the act of expiration by the falling of the chest merely. This case demonstrates the importance to life of the accessory nerves of respiration. They continued to possess a power over the diaphragm, serratus magnus anticus, trapezius, and sterno-cleido mastoideus. It is unreasonable to suppose that the patient could have survived by the mere action of the diaphragm alone, without the aid of the other muscles to enlarge the chest during its contraction."

Our author next enters into a demonstration of the truth of his views in a different manner: formerly he has been looking to the distribution of the nerves in illustration of their arrangement; but he enters here into another mode of argument, which throws additional light upon the apparatus of respiration. Our readers must recollect that the assertion has been repeatedly made, that the diaphragm performs the act of respiration when the spinal marrow is crushed in the lower part of the neck: Mr. Bell, on the contrary, affirms that this single muscle is unequal to this effect. Being attached to the ribs, it could not act forcibly without pulling down the margins of the chest.

"In as far as the diaphragm tended, by its action and by its descent, to produce a vacuum, the ribs, by their yielding to the action and their descent, would render the muscular effort nugatory: for, inasmuch as the cavities of the thorax would be enlarged in their long diameter by the descent of the diaphragm, so much would they be diminished transversely by the descent of the ribs and sternum. But when the serratus and mastoideus raise the thorax at the same moment with the contraction of the diaphragm, circumstances are materially altered: the ribs and sternum are raised against their elasticity, and consequently opposed to that state to which they would recoil even in death. The expansion of the margins of the chest increases the effect of the muscular effort of the diaphragm; the arch of that septum is contracted, and bears down; the abdominal viscera are lifted up, which, on the cessation of effort, recoil by gravitation into their position; and thus the elasticity of the ribs, and the weight of the parts opposing the muscles of inspiration, preserve the life when the muscular power of expulsion is gone."

We coincide perfectly with our author in objecting to the supposition of the diaphragm alone being capable of producing respiration; and besides we cannot conceive how expiration would be allowed to take place. The ribs and sternum being necessarily pulled down during the action of the diaphragm, they would expand by their elasticity whenever it became relaxed; and thus there would be an expansion of the chest in its transverse diameter, corresponding with the diminution produced in its long diameter by the receding of the diaphragm: that is, there would be as great a tendency to admit air as to expel it during expiration. These observations have convinced us that the sterno-cleido mastoideus, trapezius, and serratus anticus, are as necessary to respiration as the diaphragm: and when we see not only the phrenic nerve running in a peculiar manner across the symmetrical system to supply the diaphragm, but the spinal accessory and the superior thoracic nerves taking a similar course to the external muscles of respiration, we think the proof complete of their being of the same class, and destined for sustaining respiration.

These cases have been laid before the profession apparently for the purpose of inviting practitioners to observe what may be expected from prosecuting the study of the nerves. They point to several important circumstances in practice; and there is a general conclusion derived from their consideration which particularly deserves our attention.

"These cases are in illustration of the second paper, where it is shown that the nerves of the trunk, neck, and throat, are divisible into two distinct systems: the one the symmetrical system of nerves, common to all creatures, for bestowing the offices of sensation and voluntary motion, whose centre, therefore, is in the sensorium. There is a second class of superadded nerves, called the respiratory system: these are nerves which can perform their principal functions independent of the brain, and consequently of volition; for, although they be dependent for some of their functions on the efforts of the will, their principal actions may proceed during sleep, or when, from any other cause, there is an entire loss of sense and voluntary motion. The nerves of this last class are more easily excited in dying animals: they, in fact, retain life the longest, since they continue to influence the actions of respiration when sensation and volition have ceased. Thus forming a class of themselves, they are excited by sympathies which do not reach to the other nerves, and are sometimes left entire in their functions when the other class of nerves is peculiarly the seat of disturbance. These cases exhibit them very subject to derangement, and at the same time show the necessity of disentangling them anatomically, in order to distinguish the symptoms of disease which belong to them." (P. 82.)

It must be left for experience to decide whether these views will prove eminently useful in determining the nature of nervous complaints. But, while we are considering the tendency of these investigations, we must not limit our view of their importance to the direct practical benefit which they already afford. We ought rather to look forward to the results which may be expected to arise when these discoveries shall have induced practitioners in general to study the nervous system. We have ourselves derived much instruction from the labours of our author, and we hope they will soon be the means of leading practitioners to inquire into symptoms with greater precision and distinctness. In conclusion we may take the opportunity of remarking, that, as it has not been merely by the investigation of the nerves that Mr. Bell has made these discoveries, but rather by a very minute inquiry into the functions of the organs to which they are distributed, it will be found that, when practitioners renew their study of the nervous system, they will be naturally led to study the functions of the viscera,—a knowledge of which is the only true basis of pathology.

---

*Rheumatism, and some Diseases of the Heart and other internal Organs: considered in the Gulstonian Lectures, read at the Royal College of Physicians, May* 1826. By Francis Hawkins, M.D. Fellow of St. John's College, Oxford, and of the Royal College of Physicians; and one of the Physicians to the Middlesex Hospital.—8vo. pp. 144. Burgess and Hill, London, 1826.

This little volume contains the substance of the first series of Lectures delivered in the Theatre of the new College of Physicians in Pall Mall East, and we would hail it as the forerunner to some valuable contributions to the general stock of medical science from the learned body, whose splendid edifice is at once an ornament to the metropolis and a credit to the profession. We most earnestly wish that we could induce the Fellows of the College of Physicians to emulate the praiseworthy efforts of their professional brethren in Lincoln's-Inn Fields, and to afford us annually the results of that experience, and the fruits of those high talents, which are possessed by many among the members of that highly educated body. The example of the College of Surgeons is, in this respect at least, well worthy of imitation. The oldest and most experienced members of that branch of the profession have not disdained to come forward as our instructors; and they have met their reward in crowded audiences, and in increase of reputation. We do not mean to draw any invidious comparisons; but it cannot be denied that the College of Physicians have, for the most part, confided the post of lecturer to some one of the junior fellows; and, with the single exception of Dr. COOKE, (whose Croonian Lectures for 1819 were so favourably received by the public,) we do not remember any instance in which the senior fellows have lately put their shoulders to the wheel. We hope to see this altered; and indeed reports have already reached us that some change in this respect may speedily be looked for. We have in our eye at this moment many physicians, whose experience, could they be induced to give it, would be most gratefully received by a large proportion of the medical public in London; and we confidently predict that, in such a case, the theatre of the new College would be found totally inadequate to the numbers who would attend. It will probably be a matter of surprise to our country readers, when we inform them that the lecture-room of this magnificent building will with difficulty accommodate a hundred persons.

Though we have been led into this train of reflection from the perusal of the work before us, we by no means wish to insinuate that Dr. HAWKINS was undeserving of the honourable post assigned to him. On the contrary, we think that he has produced a volume useful to others and creditable to himself. It shows much learning, and is clearly and neatly put together; but, when we come to analyse it, our readers will perceive that it is chiefly occupied with speculations in pathology, which, though highly ingenious, are not to be compared, in point of value, with the results of experience in the administration of *remedies*. It is only at an hospital that such a disease as rheumatism can be properly studied; but even there, with every possible attention that can be given by an intelligent physician, many years must elapse before he has made himself conversant with all the varieties of the disease, and qualified himself to speak with confidence on those difficult and recondite points in its pathology and treatment, on which the medical world is really anxious to receive instruction. But it is time that we bring the author of the Gulstonian Lectures for 1826 more immediately before our readers; and this we shall do by giving a succinct view of the subjects discussed in each of the three lectures of which the volume is composed, selecting one or two passages out of each, as best showing the author's style of treating his subject, or as illustrating some point of particular novelty or interest.

The first lecture is occupied with speculations on the *seat* of rheumatism. The author first inquires whether the muscular texture be in any case the seat of this affection, and he argues in favour of such an opinion, from a consideration of the symptoms of rheumatism, the situation of the pain in certain cases, and the alledged appearances on dissection, as described by some foreign writers. The author very candidly states that these arguments are not conclusive, and that they only give a degree of probability to the opinion. We are next instructed that the chief seat of rheumatism is in the fibrous and tendinous structures of our frame. "It is in this quarter that rheumatism makes its most frequent invasions, exerts its most violent and extensive influence, and too often establishes a permanent dominion." We are told that Bichat was the first to collect those parts under the general name of *fibrous tissue.*

"They are frequently divided into two classes:—1. Those which serve to connect parts together; and 2. Those which divide and envelope particular organs.

"To the first class belong the tendons and ligaments, and aponeurotic expansions of tendons.

"To the second, the muscular fasciæ and enveloping aponeuroses; the periosteum; the fibrous coats of the nerves; the membranes which have on one side a serous lining, as the dura mater and pericardium; also the fibrous sheaths of the tendons and capsules of those joints which are provided with fibrous capsules, and the ligaments surrounding the other joints. To these may be added the membranes which have a mucous covering spread over them; such as the portions of the periosteum which line the palate, the nasal sinuses, and other internal cavities; and, finally, the capsules of particular organs, as the sclerotic coat and cornea of the eye, the tunica albuginea testis, and the capsules of the kidneys, ovaries, &c. All these parts, with the exception of the capsules of the solid organs, appear to be the frequent seat of rheumatic affections." (P. 19.)

The author then proceeds to notice that peculiar modification of rheumatism in which the *nerves* are principally attacked; and from analogy he is led to believe that, of the parts which belong to the nervous apparatus, the fibrous tunic, or neurilema, is the one most subject to the attacks of rheumatism. The fibro-serous membranes (the pericardium and dura mater) are then brought under review, and the fibrous capsules of certain joints, especially the hip and shoulder; and then we come to the *synovial membranes,* comprising the subcutaneous bursæ, the synovial sheaths of tendons, and the synovial capsules of joints.

"These are all of them subject to the attacks of rheumatism; and, when it occupies these structures, it may be recognised by the situation, the degree, the character, and the form of the swelling. The swelling is much greater in degree, and occurs much earlier after the commencement of the attack than that which is caused by an affection of fibrous structures. The character of the swelling is that of an elastic, circumscribed, fluctuating tumor; and its form is that of the distended synovial membrane, modified of course by the surrounding ligaments and tendons, according as these confine or admit of its free distention and protrusion.

"Another point of difference between rheumatism of the synovial and that of the fibrous membranes, which may be here briefly alluded to, is that the fever and constitutional disturbance are much greater in proportion to the degree of local inflammation in the latter, than in the former case.

"Again, it may be mentioned that, of internal organs, the heart and pericardium are chiefly prone to sympathise with an affection of fibrous structures; but the brain, and its meninges, with that of the synovial membranes." (P. 33.)

The author sums up by giving the following as the varieties of rheumatism founded on the peculiarities of anatomical structure:—1. Rheumatism of the fibrous membranes, including under it the three subdivisions of, 1, rheumatism of the tendons, fasciæ, ligaments, and muscles; 2, rheumatism of the periosteum; and, 3, rheumatism of the nerves or their fibrous sheaths.—2. The second great division of rheumatism includes the affections of synovial membranes.

The second Lecture is devoted to a description of the general and diagnostic symptoms of these forms and modifications of rheumatism, and to an explanation of their separate appearances and occasional combinations. Previous to this, however, a few remarks are offered on the exciting and predisposing causes of rheumatism, and on its intimate nature or essence. Dr. Hawkins, after expressing his distrust of Sir George Baker's theory, that neither gout nor rheumatism are really inflammations, but "that they have their seat in the exquisitely fine and slender radicles of lymphatic vessels," briefly notices the distinction between common and rheumatic inflammation; and then proceeds to the predisposition to rheumatism. This part of his subject is discussed with extraordinary brevity. We are simply told, that, whereas all circumstances which encourage corpulence and plethora predispose to gout, those which induce debility *appear* to give a tendency to rheumatism. Cautiously as this doctrine is worded, we yet think it quite untenable. The occurrence of acute rheumatism in fat and plethoric persons is far from uncommon; and accidents productive of local weakness, such as strains and contusions, are just as often the forerunners of gout as of rheumatism,—perhaps even oftener.

After giving us but one page on the predisposition to rheumatism, we were surprised by finding five pages devoted to the comparatively uninteresting subject of the Diagnosis of Acute and Chronic Rheumatism.

Rheumatism of the Fibrous Structures, as distinguished from that of the synovial membranes, next comes under notice; and, as this is the main feature of the work, we must dwell a little upon it. The author (page 84) acknowledges to have imbibed this distinction from one of the physicians to St. George's Hospital. The publication of a series of Cases of Rheumatism in some late Numbers of this Journal (July and August 1826,) will at once suggest to our readers the name of Dr. CHAMBERS as the individual here referred to. These views of rheumatism, indeed, are familiar to all who have been in the habit of attending St. George's Hospital for some years past; and, as we are informed, they have been fully developed in the lectures now in the course of delivery by Dr. Chambers, in the Theatre of Great Windmill street.* Popular language, as Dr. Hawkins remarks, has long borne testimony to such a distinction, the term *rheumatic gout* being applied to the synovial species of the disorder; and we are disposed to agree with the author, that, at the *commencement* of a rheumatic attack, the discrimination of the affected structures may be made; not perhaps, as he states, invariably, but at least very frequently. It is a matter of doubt with us whether, beyond this, the distinction is of any real value. The quantum of practical benefit resulting from this nicety in pathology, may be estimated from the author's own statement:

"Some advantage of this kind has already been obtained in the choice of remedies for the treatment of rheumatism. It has been ascertained that colchicum is almost specifically adapted for the cure of the synovial species; but that it more frequently disappoints our expectation in fibrous rheumatism, the acute form of which yields most readily to calomel and opium in considerable doses, with the interposition of occasional purgatives, and followed

* Our readers may not, perhaps, be aware that the Critical Analyses are by no means necessarily written by the Editor. We are induced to make this remark, because, connected as we are with the School of Great Windmill-street, the form of expression adopted in the above sentence might seem to savour of affectation. We beg to say, that the division of rheumatism into that affecting fibrous and synovial tissues has been adopted by Dr. CHAMBERS in his Lectures since they were first delivered; but that there are other less essential points on which Dr. HAWKINS and he are at issue. We likewise take the liberty of differing with the Reviewer with regard to the value of these pathological distinctions, which appear to us to lead to immediate and unequivocal advantages in practice.—EDITOR.

up by moderate sudorifics; and, finally, by the administration of cinchona. Topical remedies, as has been mentioned, are chiefly proper for articular rheumatism; but blisters have also a good effect in lumbago and sciatica, and deep-seated pains of the joints. Sarsaparilla and alteratives are required for chronic and cachectic cases, particularly for affections of the periosteum." (P. 88.)

When, however, we consider that the different kinds of rheumatism are frequently met with in combination, or in close succession to each other,—that both species are avowedly under the influence of the same causes, predisposing and exciting,—and that the respective remedies for these affections differ from each other more in degree than in kind, we must hesitate ere we agree with the author's concluding observation, that, "if the varieties of rheumatism are more attentively studied, a still closer adaptation of means to their cure may even yet be effected."

Some useful observations occur at page 64, on the symptoms of periosteal and nervous rheumatism, which we regret that our limits will not permit us to transcribe. We pass on, therefore, to Lecture third, which treats of the Rheumatic Affections of the Heart, and other internal organs, with a slight allusion to Rheumatic Ophthalmia. The obscurity in the symptoms of pericarditis is first touched on, and two interesting cases are related, wherein furious delirium was the leading symptom during life. The following remarks are too valuable to be passed over.

"From an examination of the cases which have been recorded of rheumatism of the heart, it appears that a large proportion of the subjects of this affection were young persons under thirty years of age: in most of them there were marks of constitutional or acquired debility: many were of a slender and delicate form, and pale and languid in their appearance. In these circumstances we have a strong confirmation of the remark, that excessive bleeding in the treatment of acute rheumatism, or any measures calculated to induce debility, increase the danger of metastasis to the heart and pericardium; and that the exhibition of bark, as soon as it can be borne with safety, is of considerable efficacy in counteracting this tendency. To the same general principle may be referred the observation, long since made, and well established, that when the pain and inflammation of external or primary rheumatism shift their situation capriciously from one limb to another, they are then most liable to be transferred to some internal organ; for the debilitating treatment before alluded to is undoubtedly calculated to give to rheumatism this migratory character." (P. 103.)

The author has taken much pains to determine what is the usual condition of the heart after repeated attacks of a rheumatic kind, and he tells us (page 115) that the tendency of this disorder is to produce simple dilatation, *without thickening*. He is inclined to suspect that, in a certain proportion of these cases of enlarged heart, the pericardium was the seat of the first attack. In cases of enlargement with hypertrophy, it is probable, he thinks, that the heart itself was primarily attacked. These speculations seem to us very open to objection. The author quotes largely from LAENNEC. We were rather surprised by the mention of one of that author's cases, in which "the heart, upon dissection, presented evident marks of having been shrunk and reduced from its former size." Dr. Hawkins appears to credit this tale, but we confess it passes our belief: the more so, as we find it brought forward, not as an insulated fact, but to support a particular practice, which Laennec had undertaken to recommend. On the means of treating these rheumatic affections of the heart, we do not find any thing in this work which is not generally known to the profession.

Pleurodyne, or rheumatism of the thoracic parietes, is next noticed. The difficulty of distinguishing this disease from internal inflammation is, according to Dr. Hawkins, "the less to be regretted, since, when rheumatism attacks the parietes of the thorax, it is so apt to be communicated to its contents, that the treatment must be nearly similar. To guard in such cases against internal inflammation, blisters should instantly be applied; since these, which are of use in any stage of the disease, have ever been found the best preventive remedy." (P. 130.)

The author will excuse us if we venture to differ from him here, both in observation and practice. We are disposed to say, that rheumatism affecting the thoracic parietes is, for the most part, a transitory and subacute affection, and by no means likely to affect the subjacent viscera. Again, instead of recommending blisters, we are in the constant habit of employing the warm bath, which we can affirm, from ample experience, to be extremely useful in this form of rheumatism. By the way, we regret that the author, who is an hospital physician, and who must have had many opportunities of using this remedy in the different kinds of rheumatism, has said so little about it, either in the way of praise or blame.

It has already been mentioned that, according to our author, metastasis to the heart chiefly occurs from diffuse rheumatism, and metastasis to the head from bursal or synovial rheumatism; cases illustrating which position were given by Dr. Chambers in the Number of this Journal for last August. With reference to rheumatic ophthalmia, a suggestion is thrown out, that, when fibrous rheumatism prevails, the sclerotic coat and iris will be found affected; and that, when the synovial membranes are the primary seat of disease, the tunica conjunctiva is the first to suffer, although the affection may subsequently extend to the deeper structures within the orbit.

In the sketch now given of Dr. Hawkins' Lectures, it has been our object to put forward those parts only which come recommended by their novelty. Our readers will, of course, understand that the more ordinary phenomena of rheumatism are also to be found in the work, rendering it a very complete treatise on this painful and still obscure disorder. We cannot better conclude than by earnestly pressing on the author a further inquiry into its phenomena, well satisfied that in his hands they will neither be overlooked, nor imperfectly investigated.

---

**On Alteration of the Nails and Disease of the surrounding Skin.* By M. Hipp. Royer Collard. (From the Répertoire Général d'Anatomie, &c. No. III.)

The relater of these cases thinks it necessary to make a few brief observations upon the structure of the parts, before he enters upon the description of the diseases incidental to them. Some anatomists have included under the term *nail* the soft parts surrounding the substance to which that term is ordinarily confined. It is absolutely necessary to determine whether our observations are confined to the nail itself, or whether they include that organ and the parts which secrete it. In the former case, we could not speak of *disease* of the nails: the nail can never be diseased, since it is not organised. But, in the latter acceptation of the term Nail, such an expression would be perfectly correct. To avoid all perplexity upon this subject, M. Collard states that he adopts the former opinion. He deems it improper to confound two such dissimilar organs. The matrix of the nail is no more the nail, than the bulb of the hair is the hair itself. M. Dupuytren, considering that the nail is produced by that portion of skin into which it sinks, and which is reflected over the edges of its circumference, has applied to it the term of *matrice de l'ongle,* (matrix of the nail.) He has called the attention of the profession to the inflammation of that part; and, after having carefully distinguished it from the disease in which the nail grows into the flesh, he has

* This article is to be regarded as a condensed translation rather than a critical analysis: perhaps it might, with more propriety, have been placed among our hospital cases.

proposed a certain mode of cure for both these species of maladies.

It has been already observed, that no disease of the nail can take place. The morbid condition must belong exclusively to the surrounding skin; but, as the nail is almost always altered in its appearance consecutively, our attention has been directed to this circumstance, and the symptom has been taken for the disease,—at least in surgical language. The intense pain which accompanies such affections, and the extreme difficulty with which they are relieved, render the subject worthy of attentive consideration.

The morbid affections of the skin surrounding the nails are always of an inflammatory nature; but the characters of such inflammation may, of course, vary with the cause producing it, and the treatment demanded will not be the same in all cases. Upon this part of the subject, M. Collard considers the clinical observations of M. Dupuytren very important. That distinguished practitioner is said to have thrown a new light upon the subject, by distinguishing the various affections of the skin of the nails into those seated in the extreme and lateral parts of the finger or toe, and into those occupying the posterior reflection which gives rise to the nail. Such an alteration of the nail, also, may exist as to injure the adjacent soft parts, or the disease may commence by inflammation of that part of the skin to which the term matrix of the nail is applied. The nail may be altered either in its substance, its form, or its direction. The cause of each of these alterations may be either external, or dependant upon derangement of the skin which secretes it. Whenever the nail is thus changed in its formation, it injures the surrounding parts, and enters into the substance of the flesh: and thus is the disease formed which is known by the name of *ongle entré dans les chairs, ongle incarné* (de Monteggia), *reserrement de l'ongle* (de Plenck), and to which M. Royer Collard has applied the term of *incarnation de l'ongle.*

With this disease most authors have confounded many other morbid affections of the skin surrounding the nails. It never occurs in the hands, and is generally seated in the great toe, on its internal angle. Wearing a tight shoe is a common exciting cause. The pain is considerable as soon as the nail enters into the neighbouring flesh; the patient walks, and even stands, with difficulty: a serous or purulent discharge soon takes place, and, if much exercised, the whole foot swells. If the disease be left to itself, the ulcer sometimes becomes cancerous, and is occasionally covered with enormous granulations. The inflammation may also extend to the periosteum, and necrosis of one or more of the phalanges may follow.

A case is related in which this disease was mistaken for gout. The removal of the nail, in the manner adopted by M. Dupuytren, entirely cured the patient.

M. Royer Collard describes at some length the various modes of practice which have been recommended by different authors, to all of which he urges various objections. He considers them all inefficacious, when the cause of the mischief arises from some alteration in the nail itself. M. Dupuytren is also of the same opinion, and for a long time he has preferred the complete evulsion of the nail to every other practice. This operation had been before performed by other surgeons, but by a very ineffectual and painful process. M. Dupuytren proceeds in the following manner:—The inflammation of the part is first relieved by appropriate treatment. The surgeon takes a strong pair of straight and very sharp scissors, one blade of which is pointed. This blade is introduced under the nail, and pushed quickly towards the middle of the root; the nail is divided into two equal portions, the incision being carried about three lines beyond its termination. The anterior part of that portion of it which keeps up the disease is then grasped by a pair of dissecting forceps, and reflected upon itself; whatever adhesions there may exist between it and the surrounding parts are successively removed, and the nail is torn away. If necessary, the operator treats the other portion of the nail in a similar manner. If the fleshy excrescences which are near the ulcer are much elevated, M. Dupuytren destroys them with the actual cautery, and thus ensures as much as possible a radical cure. The nail being thus removed, the skin underneath becomes dry, and the ulcerated portion heals in twenty-four or forty-eight hours; so that the patient, in five or six days, can resume his accustomed exercises.

In general, the nail does not grow again in old people. It is sometimes reproduced in young persons; and then, if the cause of the mischief resides in the skin which produces the nail, it is to be apprehended, in spite of every attention during its growth, that it will re-appear in an unfavourable form. Such accidents, however, are rare after the complete removal of the nail.

This mode of treatment is not applicable to every case. In 1814, Mr. Wardrop* described a species of panaris

* Medico-Chirurg. Transactions, vol. v. p. 129, "Diseases of the Toes and Fingers, by J. Wardrop." 1814.

which he designated by the name of *onychia maligna*, and which is nothing more than ulceration of the matrix of the nail. Mr. Wardrop, however, in describing this disease, of which M. Dupuytren had spoken several years ago, has confounded together many varieties of that affection, and, in consequence of this error, he has proposed the same treatment and the same common rules for cases totally dissimilar.* M. Dupuytren has clearly distinguished between the cases of "incarnation de l'ongle," and "l'ulcération de la matrice de l'ongle." Although we have observed that *disease* of the nail could not correctly be said to take place, it has been seen that the nail may be altered in its structure, in its form, and in its direction, and that, in consequence of such alterations, the surrounding soft parts would be injured. It is now, however, our object to consider those cases in which disease first occurs in the skin surrounding the nail. Such affections may either arise from an external and mechanical cause, or from the action of some particular morbid poison. When the disease does not arise from external violence, it is always difficult to detect the cause. The most common cause, in such cases, is said to be the action of the syphilitic virus, and the term *onglade*,—ulceration around the circumference of the nail,—has been applied to designate this disease. This affection, however, has not been mentioned by writers on the venereal disease. Mr. Wardrop has confounded it with common inflammation of the matrix of the nail. The subject has been very slightly noticed by M. Boyveau Laffecteur, and in an article in the Dict. des Sciences Medicales. Whether the disease does or does not depend upon the action of the syphilitic virus, it always presents particular characters:—1. It affects indifferently the nails of the feet and hands. 2. It always attacks several at the same time. 3. It sometimes commences by small ulcerations between the toes or fingers, which usually extend from their original seat around the circumference of the nails. 4. The nail is spontaneously detached. 5. It resists the anti-syphilitic treatment. M. Dupuytren has proved this fact in more than thirty cases, and he is of opinion that, however judiciously mercury may be employed, externally or internally, no benefit will be derived from its use. On the contrary, the wound generally assumes, under the exhibition of that medicine, a very unfavourable appearance, and emits a fetid odour. Mr. Wardrop,

* A reference to Mr. Wardrop's paper will show that this statement of the French writer is erroneous. That gentlemen has very clearly distinguished the two species of disease, each of which is described in a separate section. Upon some points Mr. Wardrop differs from M. Dupuytren.

however, states that he has given mercury with success. 6. As soon as the nail falls off, a simple dressing is sufficient for the cure. In such cases the wound is usually covered with a sanious or bloody suppuration, which is extremely fetid. So offensive is the odour which exhales from the feet of such patients, that it is almost impossible to remain near them. If the patient attempts to walk, or even stand, the fungi bleed. No covering can be borne on the feet; the slightest friction being excessively painful. In most patients the above symptoms occur. Sometimes the disease occupies more particularly that part of the skin which is immediately subjacent to the nail. Small tumours are then formed under the skin, from the pressure of which considerable pain arises. These tumours are of different characters: they may be either fibrous, cartilaginous, osseous, or vascular; and it is certain that their development proceeds entirely from an alteration of the skin, which is covered by the nail; for, if the tumors are removed without the skin which produces them, the latter generally again becomes diseased, and ulcerates; and, sooner or later, its entire removal is necessary.

As in this disease the skin is the part affected, our remedial means must, of course, be directed to it. If we are satisfied with only removing the nail, the seat of the disease is not destroyed, and a great number of cases have proved that it is never cured by such treatment. If we apply caustic after tearing away the nail, as Mr. Wardrop recommends, and as has been practised by Béclard, only that portion of the skin is consumed which is immediately subjacent to the nail, and all that which envelopes its root is not destroyed. M. Royer Collard knows but one successful method of treatment,—that which is employed by M. Dupuytren. The foot of the patient being fixed, and the affected toe grasped by the left hand of the surgeon, a deep and semicircular incision is made with a strait bistoury, three lines beyond, and parallel to, and surrounding, the duplicature of the skin, which supports the origin of the nail. The diseased toe being then held in its position by an assistant, the operator raises the divided skin from the root to the termination of the nail with a pair of dissecting forceps, and detaches all that portion of it which is connected with the nail, and which assisted in its production. If any fragments of naily substance still remain, they are to be successively destroyed, so that no portion of diseased tissue may be left. The operation is painful but short. The toe is lightly and simply dressed, and the patient put to bed. The leg must recline upon a pillow, and be half bent upon the thigh. In the course of three or four days, the first dressing is removed, and the wound is then usually covered with laudable pus. Simple dressings are still applied, and any granulations that may arise are kept down by caustic. If any small portions of nail are reproduced, they are to be removed, and that portion of the skin which gave rise to them destroyed with a bistoury. In about fifteen or eighteen days, the patient is able to resume his usual occupations. The disease never resists this mode of treatment, as the diseased parts are entirely removed. M. Dupuytren does not conceive that this operation is always necessary: he recommends the employment of other means, to prevent, if possible, so painful a remedy, such as antiphlogistics of all kinds, baths, rest, leeches, emollient poultices, or antisyphilitic remedies, as calomel, mercurial ointment, &c. He has sometimes successfully employed lint soaked in port wine, with one ounce to a pint of the liquor litharg. acet. In support of these opinions, many cases are related from the practice of the Hotel Dieu. Some of them are instances of simple incarnation of the nail; others of affections of the skin forming the matrix of the nail. Many are complications of both these species of disease. We have selected some of the most interesting examples:—

Case I. *The Nail penetrating the Flesh: Removal of the external Half of the Nail.*

Jacques Roussin, seventeen years of age, of a strong constitution, was admitted into the Hôtel Dieu, June 18th, 1821. For the last six months he had worn very tight and thick shoes, by which his feet were much compressed. The external angle of the nail of the great toe was bent inwards, and penetrated the flesh which covered it, and which was much swollen. The part was red and painful, and the lameness gradually increased. The fleshy excrescence of the part confined by the other toes, and pressed upon in every direction by the shoe, became hard, callous, and of a whitish colour. The patient continued to work, although he suffered considerably. A small wound at length taking place, which occasionally bled and discharged a small quantity of pus, he determined to enter the hospital.

After a few days' rest, and the use of baths and some emollient applications, M. Dupuytren removed that portion of the nail which was covered by the surrounding flesh. The nail was divided down the middle with a pair of strong straight scissors; the external half was grasped with dissecting forceps, reflected upon itself, and torn away. The callous skin which covered it was removed with the scissors. But a small quantity of blood was lost. The patient was put to bed, and the part simply dressed. In four days he left the hospital cured; but he was desired to wear larger shoes, and to cover the toe with a pledget and simple ointment.

Case II. *Removal of the Matrix of the Nail; Cauterisation of the Skin.*

M. Simon, twenty-three years of age, of good constitution, was admitted the 23d February, 1819. The great toe of the left foot discharged a greyish and fetid matter. The nail had partly fallen off, and the remaining portion of it penetrated into the flesh, and maintained a constant irritation and considerable discharge. When the patient attempted to walk, the toe bled. About six weeks before, a piece of wood had fallen upon her foot: inflammation and suppuration of the toe ensued, and a portion of the nail was detached, which was removed by a surgeon, who allowed the root of it to remain, as it still adhered to the surrounding parts. Notwithstanding the removal of the separated part of the nail, the distressing symptoms still continued, and she requested admission into the hospital.

The inflammatory symptoms were first relieved by appropriate treatment, and M. Dupuytren then proposed to remove the nail entirely, and to cauterise the diseased skin, so as to convert the ulcer into a simple wound. He removed with a bistoury all the skin which surrounded the nail, and the nail itself, and then cauterised with a red hot iron the bleeding surface. The operation was short, but excessively painful. The wound was dressed with simple ointment and lint, and a light bandage was applied. The dressings were removed five days after the operation: suppuration had commenced, and the parts destroyed by the cautery had separated. Digestive ointment was applied. In about a month from the performance of the operation, the wound was entirely healed, and the patient was dismissed. She was recommended to remain as quiet as possible for some days.

Case III. *Disease of the Matrix of the Nail; Removal of the Skin, and Cauterisation of the Part.*

In June 1822, a young girl, affected with inflammation of the skin which serves for the reception of the nail, was admitted into the Hôtel Dieu. The disease had existed for years. M. Dupuytren removed with a bistoury all the surrounding skin and the nail itself, which had not penetrated the adjacent soft parts. Fifteen days after the operation, a small portion of naily substance being reproduced, it was removed with dissecting forceps, and M. D. cauterised the bottom of the fresh wound which he had formed, with a piece of lint soaked in a solution of nitrate of mercury. At the end of twenty days, the cicatrix was complete and remarkably firm, and the patient left the hospital.

Several other cases are given, illustrative of the efficacy of the treatment pursued by M. Dupuytren. The Atlas contains also some engravings of the two species of the disease described in the paper.

The principal points which are insisted upon in the above paper are—

1st. The necessity of distinguishing that affection of the toe or finger which arises from the entrance of the nail into the surrounding flesh, from that disease which is not very uncommon in the same parts, and the cause of which is a diseased condition of the secreting organ of the nail.

2d. That each of these two maladies requires a different mode of practice.

The profession must feel indebted to M. Dupuytren for the attention he has devoted to so painful, although apparently unimportant, a disease. But we must suggest to M. Royer Collard, that neither the views which he promulgates in his paper respecting the nature of the disease, nor the treatment, which he considers peculiar to M. Dupuytren, can lay claim to originality. We have thought, however, that it would be of use to bring the subject before our readers, because, although the views contained in this paper have long been known to some English surgeons, still we believe that they are not so generally diffused as their importance deserves.

---

*Medical Botany; or, Illustrations and Descriptions of the Medicinal Plants of the London, Edinburgh, and Dublin Pharmacopœias, with those lately introduced into Medical Practice: comprising their Generic and Specific Characters; English, Provincial, and Foreign Appellations; a copious List of Synonyms; Botanical Descriptions; Natural History; Physical, Chemical, and Medical Properties and Uses. Including also a Popular and Scientific Description of Poisonous Plants, particularly those that are indigenous to Great Britain and Ireland; with Figures coloured from Nature. The whole forming a complete System of Vegetable Toxicology and Materia Medica.* By JOHN STEPHENSON, M.D. Graduate of the University of Edinburgh; and JAMES MORSS CHURCHILL, Esq. Surgeon, Fellow of the Medico-Botanical Society of London. Nos. I. and II. (to be continued monthly.)—8vo. John Churchill, London, 1827.

THE two first Numbers only of this work have appeared; but we have no hesitation in saying that, if the subsequent parts resemble those before us in their execution, the undertaking will merit the countenance of the profession. That a correct set of engravings of medicinal plants is a desideratum, cannot be doubted, as at present we have none which are even tolerably good; and it is the desire to give publicity to the attempt now made to remedy this evil which induces us to depart from a general rule, by taking notice of an *indigenous* periodical.

Each Number contains four plates; and the engravings are really very prettily executed.

In a work of this nature, we may say, without derogating from the character of the author, that the value depends principally on the fidelity of the drawings; and this is the point, too, in which most originality may be shown. The description of the plants, and the details of their properties, must necessarily be principally compiled from preceding writers, although there is abundant room for selection in the materials.

The plan is as follows:—we have first the name of the plant, with its place in the classifications of Linnæus and Jussieu; its generic and specific characters and synonyms. Next comes the locality; a more particular description, with reference to the plate; the qualities, chemical composition, the medical properties and uses; the effects when the substance acts as a poison; the doses, officinal preparations, and formulæ.

Having thus introduced the Medical Botany to our readers, we have only left to wish the editors success in an undertaking which we think so well calculated to be of use to the profession.

